# Supplementary material for: AP-4 vesicles contribute to spatial control of autophagy via RUSC-dependent peripheral delivery of ATG9A
Source: Nat Commun. 2018 Sep 27;9:3958. doi: 10.1038/s41467-018-06172-7 (PMC6160451; doi:10.1038/s41467-018-06172-7)
Supplement: Supplementary file 1 — Description of Additional Supplementary Files [file 41467_2018_6172_MOESM1_ESM.pdf]

Description of Additional Supplementary Information

File Name: Supplementary Data 1

Description: Dynamic Organellar Maps profiles and MR plot shift analysis. Processed mass spectrometry data used to produce the Dynamic Organellar Maps of wildtype, AP4B1 knockout and AP4E1 knockout cells, related to Fig. 1. Sheet 1 shows normalised SILAC ratio profiles used to generate the maps. Sheet 2 shows the data used to generate the MR plot shown in Fig. 1e.

File Name: Supplementary Data 2

Description: Dynamic Organellar Maps marker protein neighbourhood analysis. Results of the neighbourhood analysis performed on the Organellar Maps data shown in Supplementary Data 1, related to Fig. 1.

File Name: Supplementary Data 3

Description: AP-4 vesicle profiling MS data. Processed mass spectrometry data used in the comparative profiling of vesicle fractions from control and AP-4-depleted cells, related to Fig. 2b and Supplementary Fig. 2a.

File Name: Supplementary Data 4

Description: AP-4 knockouts proteomic analyses and TEPSIN-GFP IP MS data. Processed mass spectrometry data, related to Fig. 2d, f and Supplementary Fig. 1c and 2e. Sheet 1 shows the data used for global proteomic analysis of AP-4 knockout HeLa cells. Sheet 2 shows the data used for membrane fraction analysis of AP-4 knockout cells. Sheet 3 shows the data from the sensitive TEPSIN-GFP immunoprecipitations and sheet 4 shows the data from the conventional TEPSIN-GFP immunoprecipitations.

File Name: Supplementary Data 5

Description: AP-4 BioID MS data. Processed mass spectrometry data and analysis from the AP-4 BioID experiments, related to Fig. 2e and Supplementary Fig. 2b-d. Sheet 1 shows the LFQ intensity data. Sheet 2 shows the data with control compression. Sheets 3-6 show the data following transformation and imputation, used to generate the volcano plots for each AP-4 subunit.
