# Supplementary material for: AP-4 vesicles contribute to spatial control of autophagy via RUSC-dependent peripheral delivery of ATG9A
Source: Nat Commun. 2018 Sep 27;9:3958. doi: 10.1038/s41467-018-06172-7 (PMC6160451; doi:10.1038/s41467-018-06172-7)
Supplement: Supplementary file 8 — Supplementary Information [file 41467_2018_6172_MOESM8_ESM.pdf]

AP-4 vesicles contribute to spatial control of autophagy via RUSC-dependent peripheral delivery of ATG9A

Davies et al.

Supplementary Information

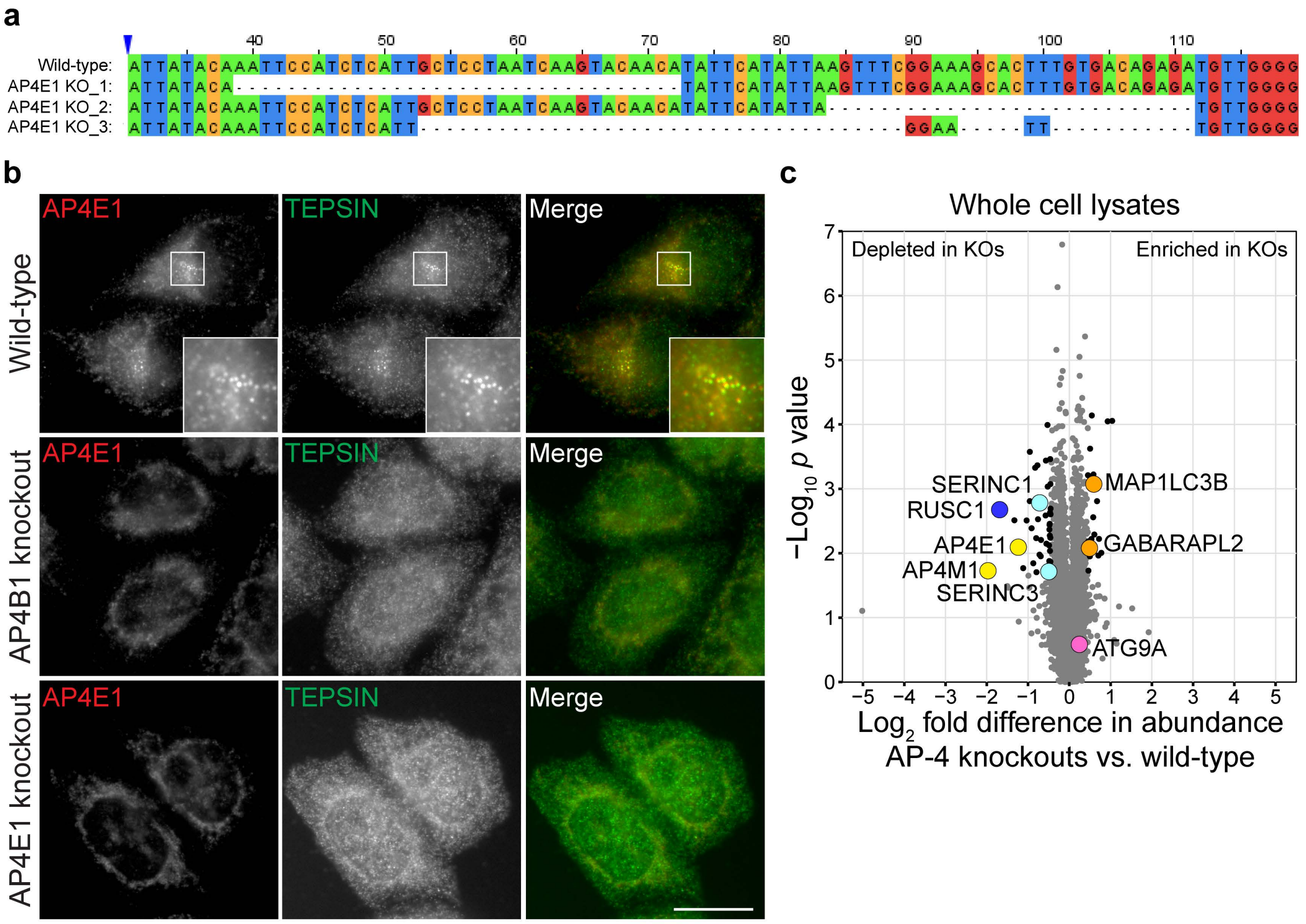

Supplementary Figure 1. Characterisation of AP-4 knockout (KO) HeLa cells. (**a**) Multiple sequence alignment of wild-type *AP4E1* exon 6 and the mutant sequences recovered from *AP4E1* KO clone x6C3. Allele 1 (34 bp deletion) was recovered seven times, allele 2 (28 bp deletion) six times and allele 3 (compound 53 bp deletion) four times. The alignment shows only the area of exon 6 surrounding the mutations (the remainder of the exon 6 sequence was identical in the mutants and the wild-type). The nucleotides are numbered based on the wild-type allele, starting from 1 for the first nucleotide of exon 6. All mutant alleles result in frameshift and the introduction of a premature termination codon in exon 6. Sequencing data for the *AP4B1* KO clone were published previously[1].
(**b**) Widefield immunofluorescence imaging of AP4E1 and TEPSIN in wild-type and AP-4 KO HeLa cells. Note that the AP4E1 antibody has some background staining; the signal that is specific to AP4E1 is the cluster of puncta in the perinuclear region of the cell (inset). AP-4 KO resulted in the concomitant loss of TEPSIN puncta from this region, indicative of loss of AP-4 function. Scale bar: 20 µm.
(**c**) Global proteome analysis of AP-4 KO HeLa cells. Whole cell lysates from *AP4B1* and *AP4E1* KO HeLa cells were analysed by SILAC-based quantitative mass spectrometry, in comparison to lysates from heavy SILAC-labelled wild-type cells (in triplicate for each KO line). >6,500 proteins were quantified. Data were analysed with a two-tailed one sample t-test. Significance cut-offs were defined as $p \leq 0.02$ and a minimum absolute fold change ($\log_2$) of 0.45 (proteins marked with black or coloured circles meet these criteria, except ATG9A), with an estimated FDR of 25%.

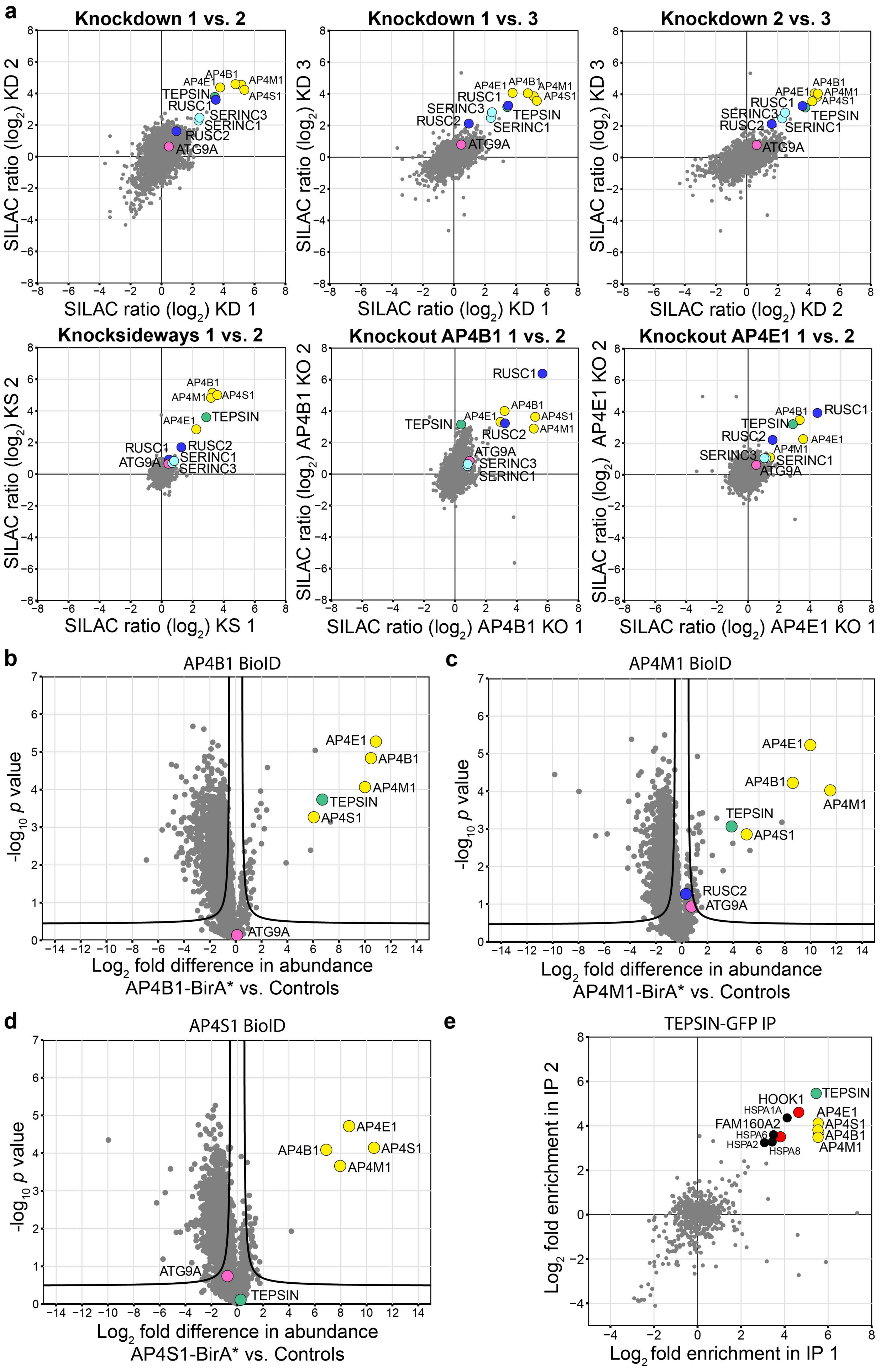
a
Knockdown 1 vs. 2
SILAC ratio (log2) KD 2
SILAC ratio (log2) KD 1
AP4B1
AP4E1
AP4M1
AP4S1
TEPSIN
RUSC1
SERINC3
SERINC1
RUSC2
ATG9A
Knockdown 1 vs. 3
SILAC ratio (log2) KD 3
SILAC ratio (log2) KD 1
Knockdown 2 vs. 3
SILAC ratio (log2) KD 3
SILAC ratio (log2) KD 2
Knocksideways 1 vs. 2
SILAC ratio (log2) KS 2
SILAC ratio (log2) KS 1
Knockout AP4B1 1 vs. 2
SILAC ratio (log2) AP4B1 KO 2
SILAC ratio (log2) AP4B1 KO 1
Knockout AP4E1 1 vs. 2
SILAC ratio (log2) AP4E1 KO 2
SILAC ratio (log2) AP4E1 KO 1
b
AP4B1 BioID
-log10 p value
Log2 fold difference in abundance
AP4B1-BirA* vs. Controls
c
AP4M1 BioID
Log2 fold difference in abundance
AP4M1-BirA* vs. Controls
d
AP4S1 BioID
Log2 fold difference in abundance
AP4S1-BirA* vs. Controls
e
TEPSIN-GFP IP
HOOK1
HSPA1A
FAM160A2
HSPA6
HSPA2
HSPA8
Log2 fold enrichment in IP 2
Log2 fold enrichment in IP 1

Supplementary Figure 2. Orthogonal proteomic approaches to identify AP-4 cargo and accessory proteins. (**a**) SILAC quantitative proteomic profiling of AP-4-depleted vesicle fractions, with AP-4 depletion by knockdown (three replicates), 60 minutes knocksideways (two replicates), or knockout of *AP4B1* or *AP4E1* (two replicates each). This figure shows the nine individual experiments that were combined in Fig. 2b. SILAC ratios (wild-type/AP-4-depleted) from replicate experiments are plotted against each other. Proteins that are lost from the vesicle-enriched fraction in the absence of AP-4 are found in the top right sectors of the plots. The different depletion approaches act on different time scales, and hence have subtly different effects on vesicle composition. All methods show very similar overall trends though. (**b-d**) BioID with AP4B1 (**b**), AP4M1 (**c**) and AP4S1 (**d**) subunits. Comparison of protein abundance in affinity purifications of biotinylated proteins from HeLa cells stably expressing BirA*-tagged AP-4 subunits and control cell lines (HeLa, HeLa BirA* and HeLa GFP-BirA), analysed by label-free quantitative mass spectrometry (MS). Each experiment was performed in triplicate and the control dataset was compressed to the three highest LFQ intensities per protein (i.e. high stringency). Data were analysed with a two-tailed t-test: volcano lines indicate the significance threshold (FDR=5%). BioID is a proximity-based method, so different results were expected for the different AP-4 subunits. (**e**) Co-immunoprecipitation of AP-4 complexes via anti-GFP from HeLa cells stably expressing TEPSIN-GFP, using conventional immunoprecipitation conditions. The experiment was performed in duplicate and proteins were quantified by SILAC-based MS, relative to mock immunoprecipitations from parental HeLa controls. Proteins that co-purify with TEPSIN-GFP are: the four AP-4 subunits, heat shock proteins, HOOK1 (Protein Hook homolog 1), and FAM160A2 (FTS and Hook-interacting protein). HOOK1 and FAM160A2 are two members of the FHF complex and FAM160A2 was previously identified in an affinity purification of the AP-4 complex[2].

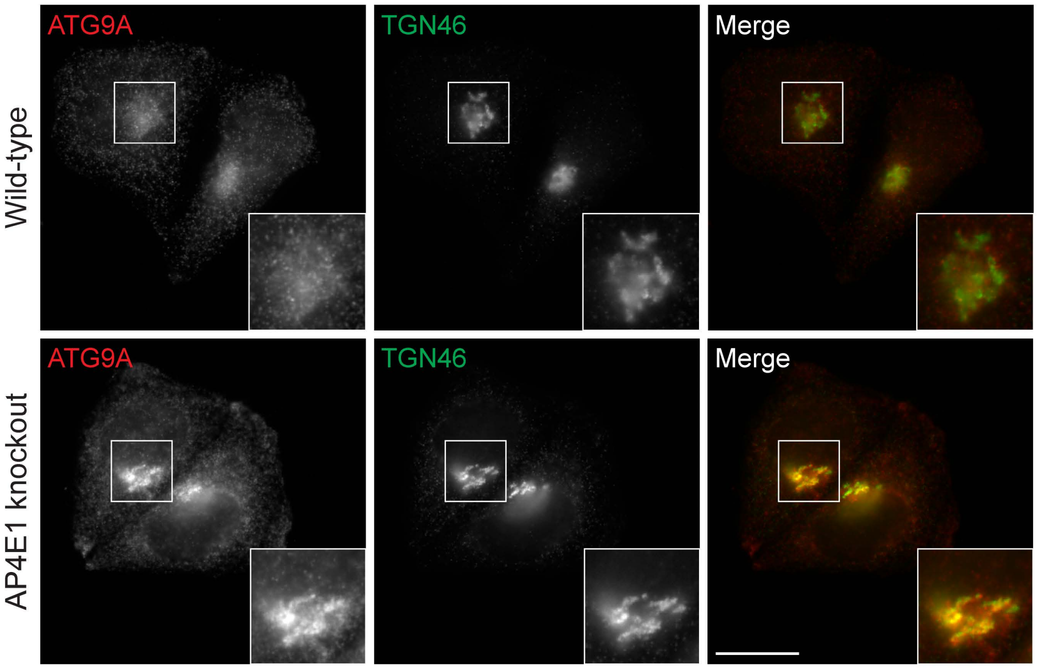

Supplementary Figure 3. ATG9A accumulates at the trans-Golgi network in *AP4E1* knockout Hela cells.
Widefield imaging of anti-ATG9A and anti-TGN46 in wild-type and *AP4E1* knockout HeLa cells.
Scale bar: 20 µm.

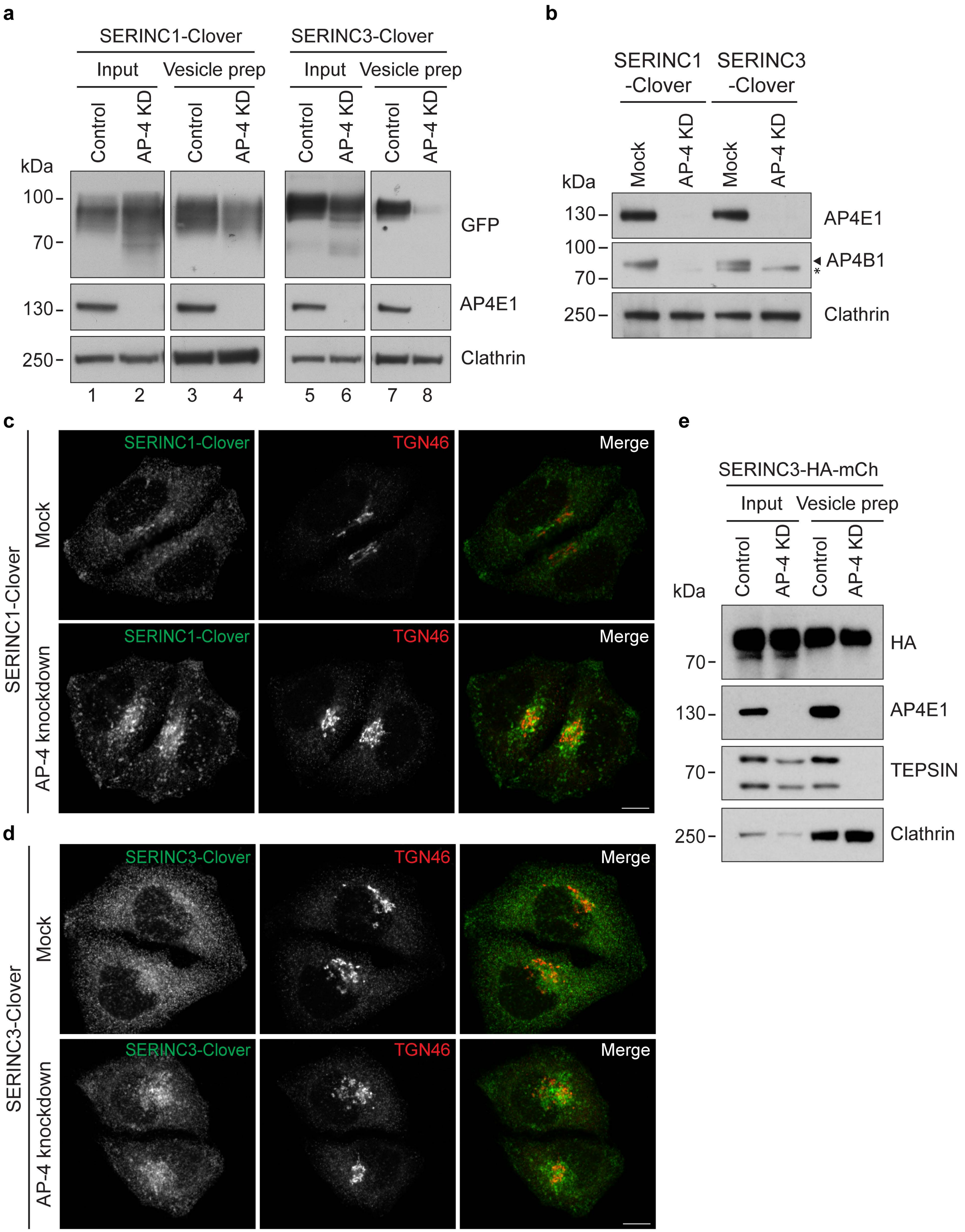
a
SERINC1-Clover
SERINC3-Clover
Input
Vesicle prep
Input
Vesicle prep
Control
AP-4 KD
Control
AP-4 KD
Control
AP-4 KD
Control
AP-4 KD
kDa
100
70
130
250
GFP
AP4E1
Clathrin
1
2
3
4
5
6
7
8
b
SERINC1 -Clover
SERINC3 -Clover
Mock
AP-4 KD
Mock
AP-4 KD
kDa
130
100
70
250
AP4E1
AP4B1
*
Clathrin
c
SERINC1-Clover
Mock
AP-4 knockdown
SERINC1-Clover
TGN46
Merge
d
SERINC3-Clover
Mock
AP-4 knockdown
SERINC3-Clover
TGN46
Merge
e
SERINC3-HA-mCh
Input
Vesicle prep
Control
AP-4 KD
Control
AP-4 KD
kDa
70
130
70
250
HA
AP4E1
TEPSIN
Clathrin

Supplementary Figure 4. Endogenously tagged SERINC1- and SERINC3-Clover are mistrafficked in AP-4-depleted HeLa cells. (**a**) Vesicle-enriched fractions were prepared from HeLa SERINC1-Clover and HeLa SERINC3-Clover cells, with and without knockdown of AP-4 by siRNA. Western blots of input material and vesicle-enriched fractions; Clathrin heavy chain, loading control. As expected, SERINC1- and SERINC3-Clover were depleted from the vesicle-enriched fractions prepared from AP-4 knockdown cells, indicating that the tagged SERINCs are trafficked like the endogenous proteins. (**b**) Western blots of whole cell lysates from HeLa SERINC1- and SERINC3-Clover cells either mock transfected or transfected with siRNA to knock down AP-4, prepared in parallel to the cells used for imaging in the experiments shown in Fig. 5; Clathrin heavy chain, loading control. Note the AP4B1 antibody labels a non-specific band which is marked by the asterisk. (**c**) Confocal imaging of anti-GFP and anti-TGN46 in HeLa SERINC1-Clover cells, with or without AP-4 knockdown (control was mock transfected without siRNA). Scale bar: 10 µm. (**d**) HeLa SERINC3-Clover cells analysed as in **c**. SERINCs accumulate in the perinuclear region of AP-4-depleted cells, but do not show obvious overlap with TGN46. (**e**) Vesicle-enriched fractions were prepared from HeLa cells stably overexpressing SERINC3-HA-mCherry, with and without knockdown of AP-4 by siRNA. Western blots of input material and vesicle-enriched fractions; Clathrin heavy chain, loading control. Loss of TEPSIN from the vesicle-enriched fraction prepared from the AP-4 knockdown cells confirmed loss of AP-4 function. Unlike endogenously tagged SERINCs, overexpressed SERINC3-HA-mCherry was not depleted from the AP-4 knockdown vesicle-enriched fraction.

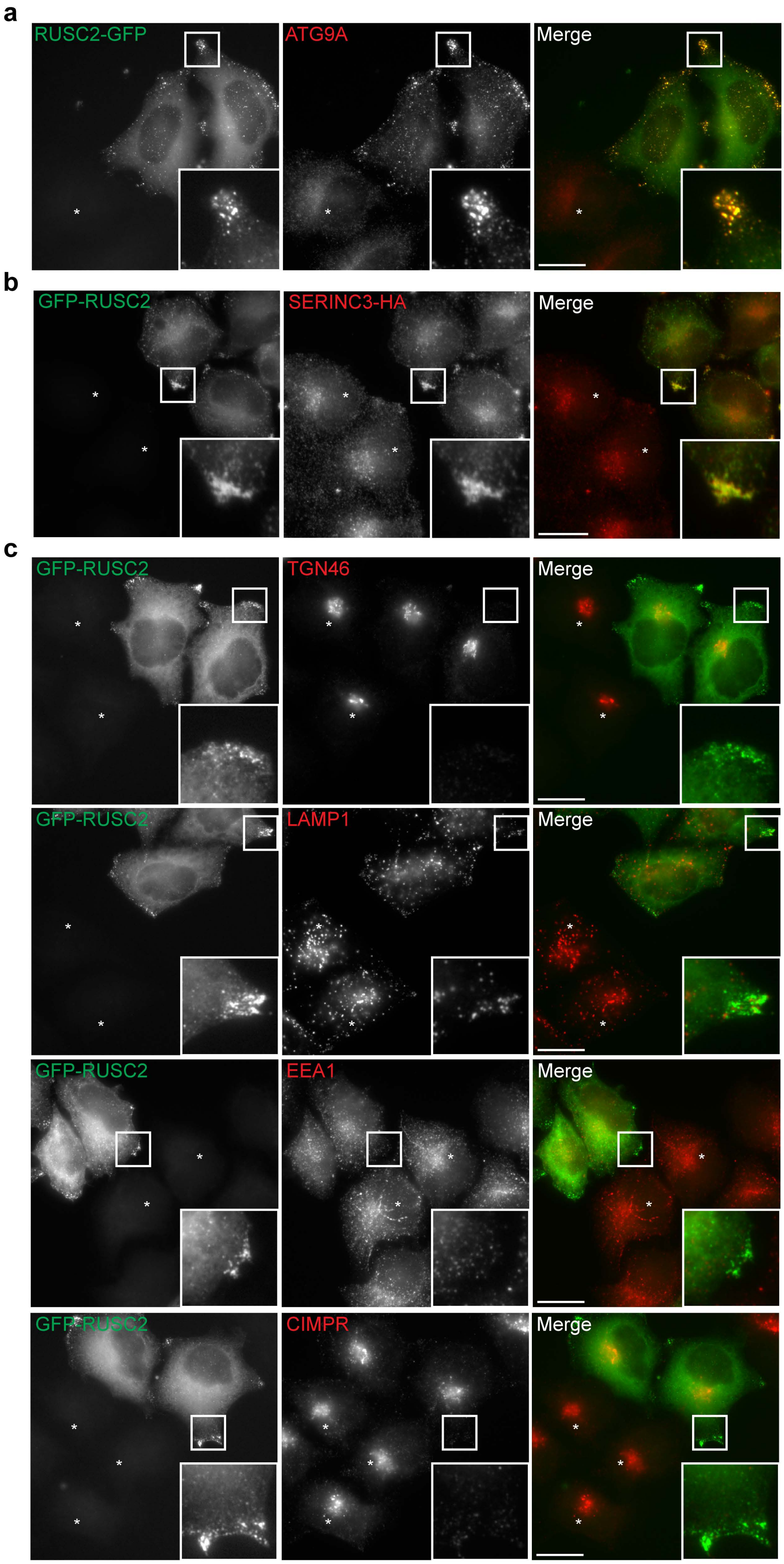

a
RUSC2-GFP
ATG9A
Merge
b
GFP-RUSC2
SERINC3-HA
Merge
c
GFP-RUSC2
TGN46
Merge
GFP-RUSC2
LAMP1
Merge
GFP-RUSC2
EEA1
Merge
GFP-RUSC2
CIMPR
Merge

Supplementary Figure 5. Overexpression of RUSC2 results in accumulation of AP-4 cargo proteins at the periphery of the cell. (**a**) Widefield imaging of HeLa cells stably expressing RUSC2-GFP, mixed on coverslips with parental HeLa cells (marked with asterisks), labelled with anti-ATG9A. The insets show accumulation of RUSC2-GFP- and ATG9A-positive puncta at the periphery of the cell. Scale bar: 20 µm. (**b**) Widefield imaging of a mixed population of HeLa cells stably expressing GFP-RUSC2 and HA-tagged SERINC3, labelled with anti-HA. Cells only positive for HA-tagged SERINC3 are marked by an asterisk. The insets show accumulation of GFP-RUSC2- and HA-positive puncta at the periphery of the cell. Scale bar: 20 µm. (**c**) Widefield imaging of HeLa cells stably expressing GFP-RUSC2, mixed on coverslips with parental HeLa cells (marked with asterisks), labelled with anti-TGN46, anti-LAMP1, anti-EEA1 or anti-CIMPR. The insets show accumulation of GFP-RUSC2-positive puncta at the periphery of the cells, without redistribution of the co-labelled protein, demonstrating that the effect of RUSC2 overexpression is specific for AP-4 cargo proteins. Scale bar: 20 µm.

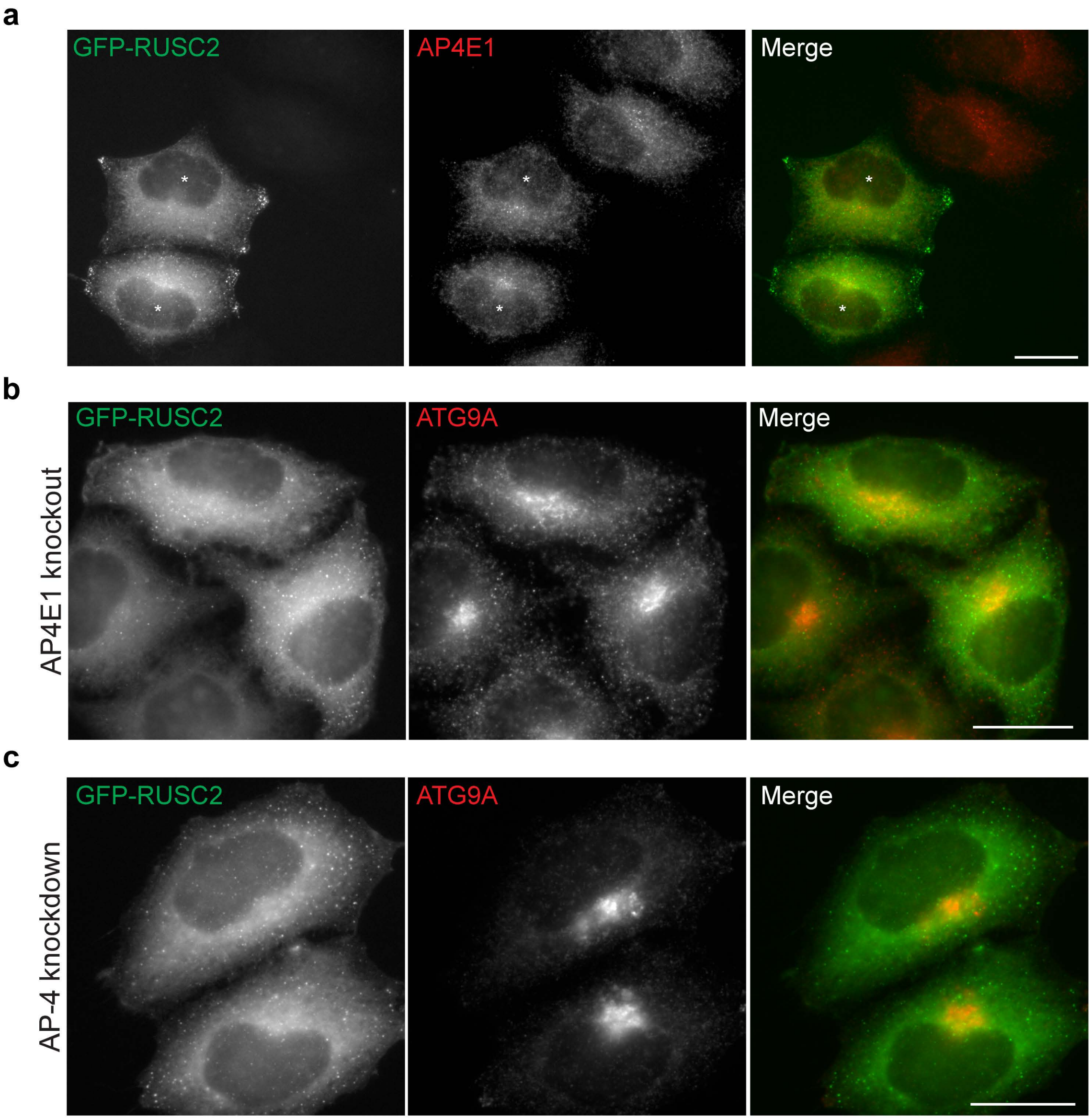

Supplementary Figure 6. The effect of RUSC2 overexpression on the localisation of ATG9A is AP-4-dependent. (**a**) Widefield imaging of HeLa cells stably expressing GFP-RUSC2 (marked with asterisks), mixed on coverslips with parental HeLa cells, labelled with anti-AP4E1. Overexpression of RUSC2 does not alter the distribution of AP-4 itself. Scale bar: 20 µm. (**b**) Widefield imaging of AP4E1 knockout HeLa cells stably expressing GFP-RUSC2, labelled with anti-ATG9A. GFP-RUSC2 puncta do not accumulate at the cell periphery, nor do they colocalise with ATG9A. Scale bar: 20 µm. (**c**) Widefield imaging of HeLa cells stably expressing GFP-RUSC2 treated with siRNA to knock down AP-4, labelled with anti-ATG9A. As above, GFP-RUSC2 puncta neither accumulate at the cell periphery, nor colocalise with ATG9A. Scale bar: 20 µm.

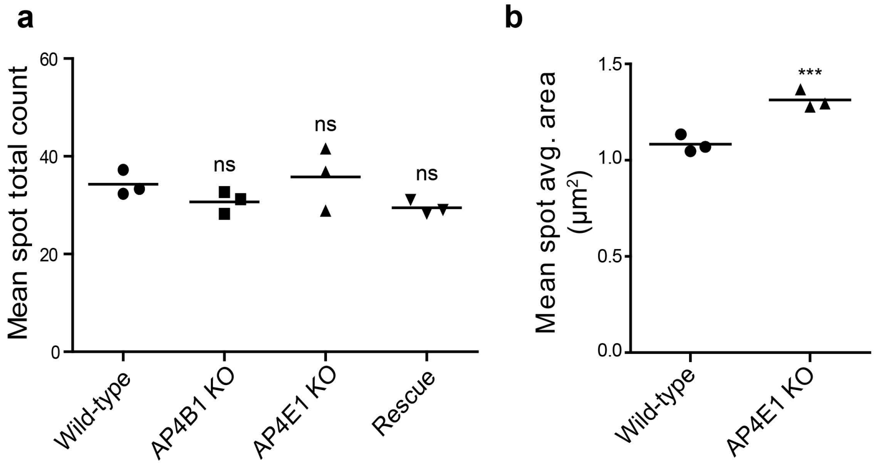

Supplementary Figure 7. AP-4 knockout HeLa cells have enlarged autophagosomes. Wild-type, *AP4E1* knockout, *AP4B1* knockout, and *AP4B1* knockout HeLa cells rescued with stable expression of AP4B1 were starved for two hours in EBSS and then fixed and labelled with anti-LC3B. LC3B puncta were quantified using an automated microscope (data from the same experiment as that shown in Fig. 8d). The experiment was performed in biological triplicate (mean indicated, n=3) and more than 500 cells were scored per cell line in each repeat. Data were analysed by one-way ANOVA with Dunnett's Multiple Comparison Test for significant differences to the wild-type: ns $p > 0.05$; *** $p \leq 0.001$. (**a**) Quantification of the total number of LC3B-positive spots per cell in each cell line showed no significant difference between the cell lines. (**b**) Quantification of the apparent size (in µm^2) of LC3B puncta in wild-type and *AP4E1* knockout cells. The average spot area (in µm^2) was significantly larger in the *AP4E1* knockout cells, as was observed for the *AP4B1* knockout line (Fig. 8d).

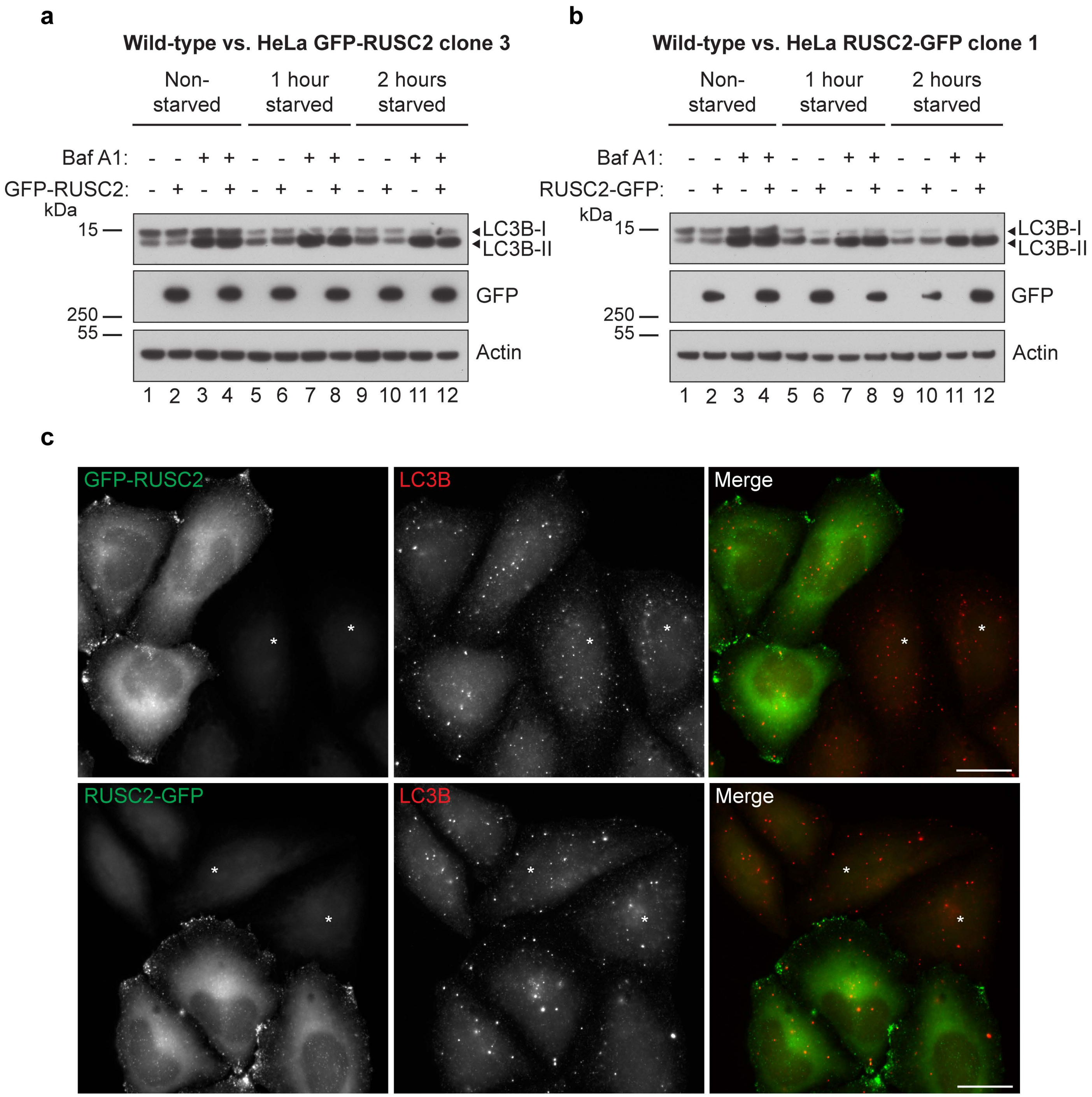

Supplementary Figure 8. Overexpression of RUSC2 does not affect LC3B levels. (**a**) Western blots of whole-cell lysates from wild-type HeLa cells and HeLa cells stably expressing GFP-RUSC2, cultured in full medium or starved for one or two hours in EBSS, with or without the addition of bafilomycin A1 (100 nM, 2 hours); Actin, loading control. (**b**) Western blots of whole-cell lysates from wild-type HeLa cells and HeLa cells stably expressing RUSC2-GFP, as described in **a**. The wild-type samples are the same as the samples shown in **a**. (**c**) Widefield imaging of HeLa cells stably expressing GFP-RUSC2 or RUSC2-GFP, mixed on coverslips with parental HeLa cells (marked with asterisks), cultured in full medium, labelled with anti-LC3B. Scale bar: 20 µm.

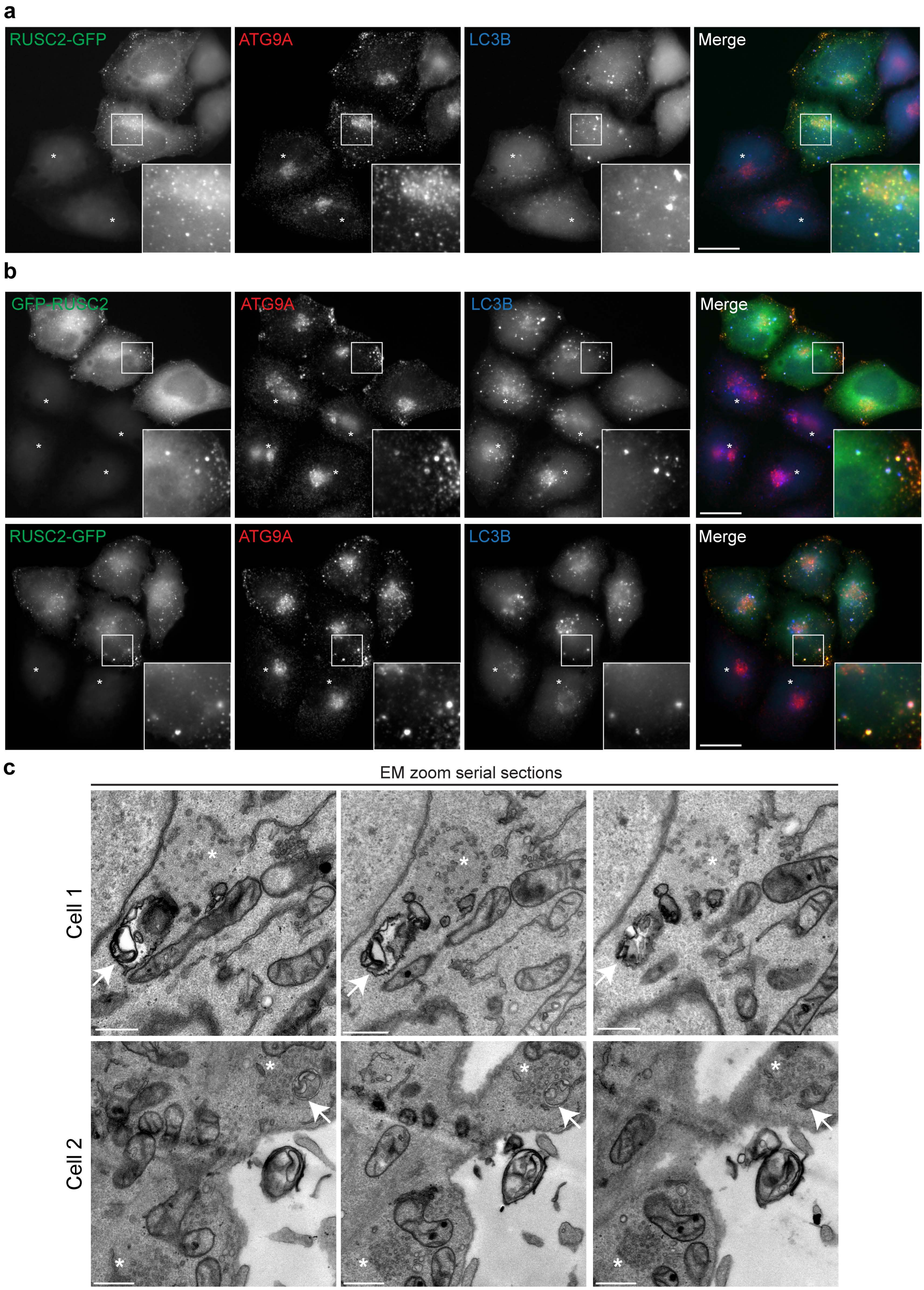
a
RUSC2-GFP
ATG9A
LC3B
Merge
b
GFP-RUSC2
ATG9A
LC3B
Merge
RUSC2-GFP
ATG9A
LC3B
Merge
c
EM zoom serial sections
Cell 1
Cell 2

Supplementary Figure 9. RUSC2- and ATG9A-positive vesicles closely associate with autophagosomes in starved cells. (**a**) Widefield imaging of HeLa cells stably expressing RUSC2-GFP, mixed on coverslips with parental HeLa cells (marked with asterisks), starved for two hours in EBSS, labelled with anti-ATG9A and anti-LC3B. The insets show RUSC2-GFP- and ATG9A-positive puncta in very close proximity to LC3B puncta. Scale bar: 20 µm.
(**b**) Widefield imaging of HeLa cells stably expressing GFP-RUSC2 or RUSC2-GFP, mixed on coverslips with parental HeLa cells (marked with asterisks), starved for two hours in EBSS with 100 nM bafilomycin A1, labelled with anti-ATG9A and anti-LC3B. Scale bar: 20 µm.
(**c**) Serial sections from the correlative light and electron microscopy (CLEM) of HeLa cells stably expressing RUSC2-GFP, starved for two hours in EBSS with 100 nM bafilomycin A1, shown in Fig. 9c. The clusters of small uncoated vesicular and tubular structures that correspond to RUSC2-GFP puncta are marked with asterisks and autophagosomes are marked with arrows. The middle image of each set of three is the zoom image shown in Fig. 9c. Scale bar: 500 nm.

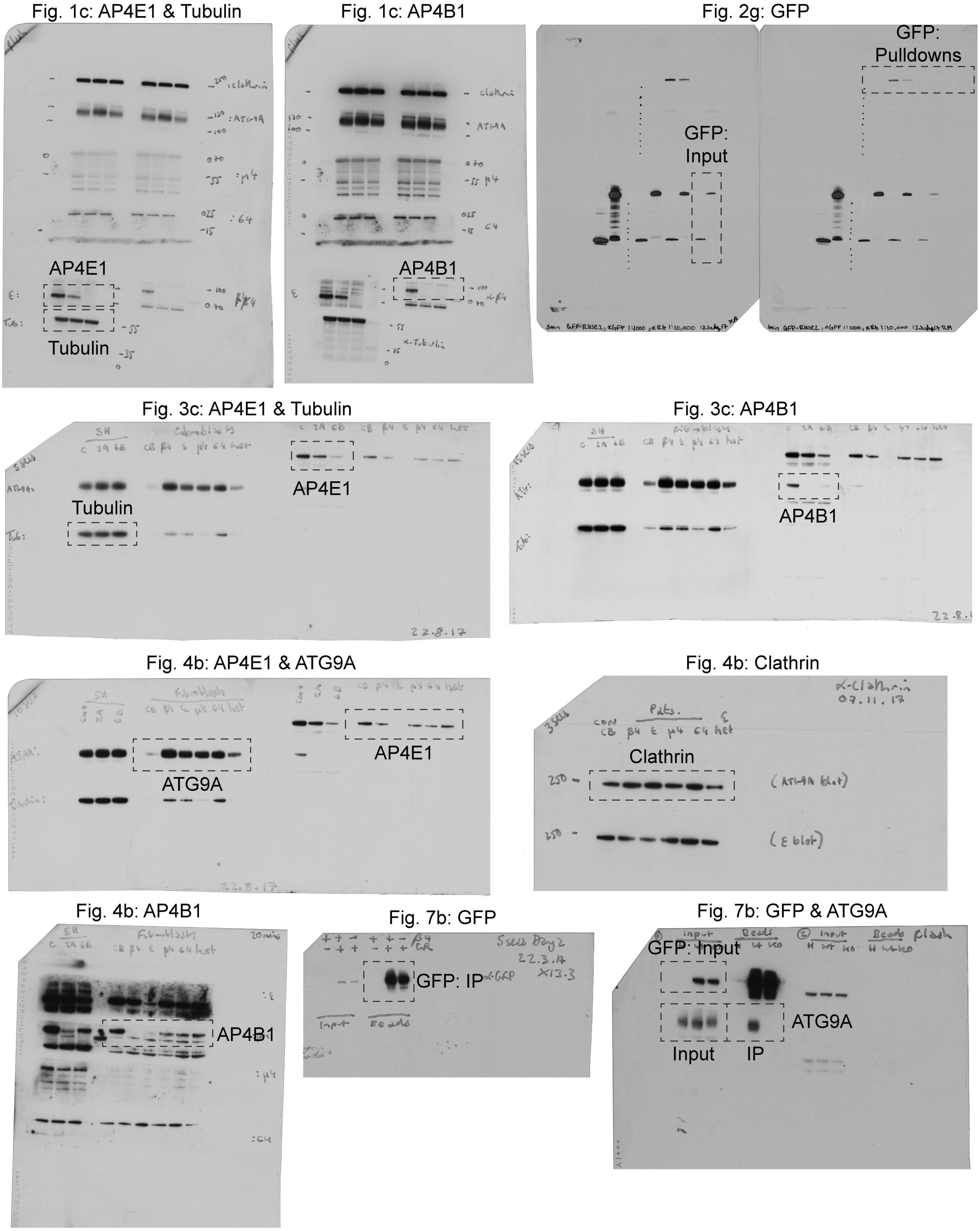

Supplementary Figure 10. Uncropped scans of Western blots shown in Fig. 1, 2, 3, 4 and 7. The relevant lanes and the figures in which they appear are indicated on each blot.

Fig. 8a, b: LC3B

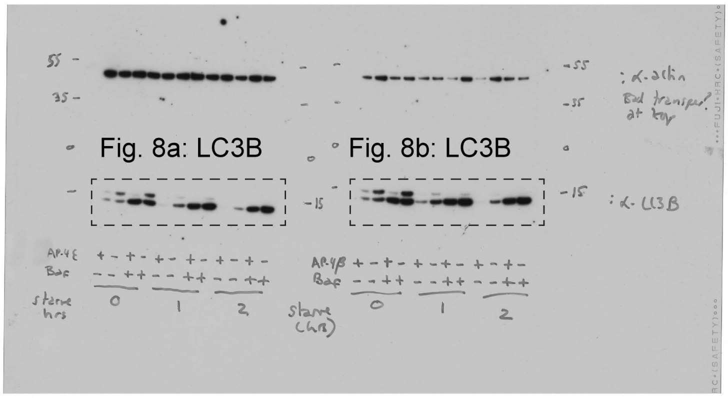

Fig. 8a: AP4B1

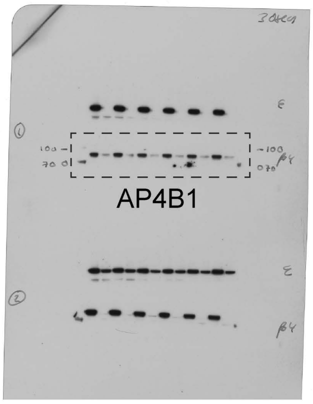

Fig. 8a, b: AP4E1 & Clathrin

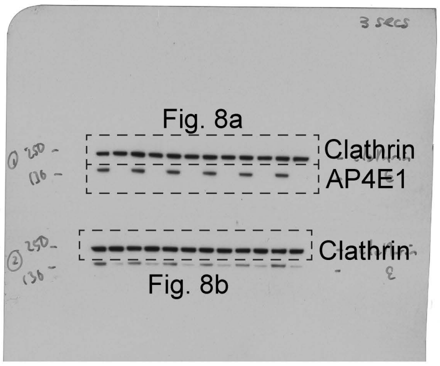

Fig. 8g Full medium: LC3B

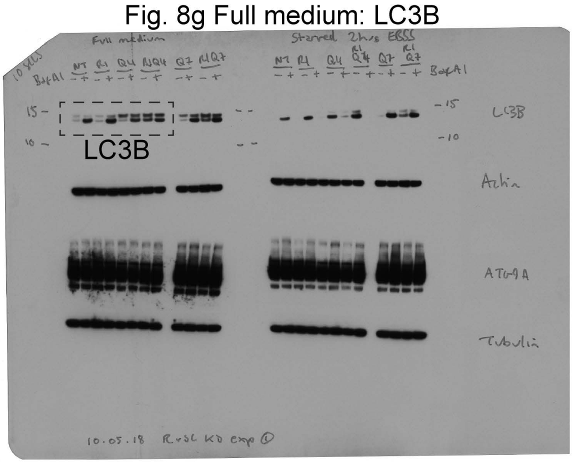

Fig. 8b: AP4E1

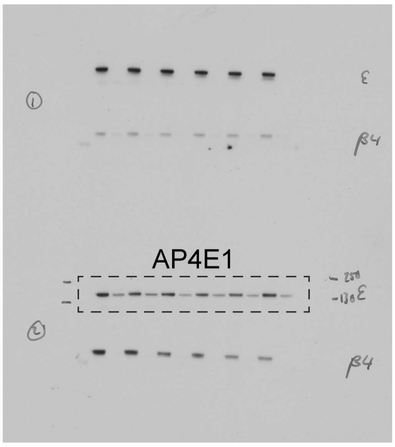

Fig. 8g 2 hours starved: LC3B

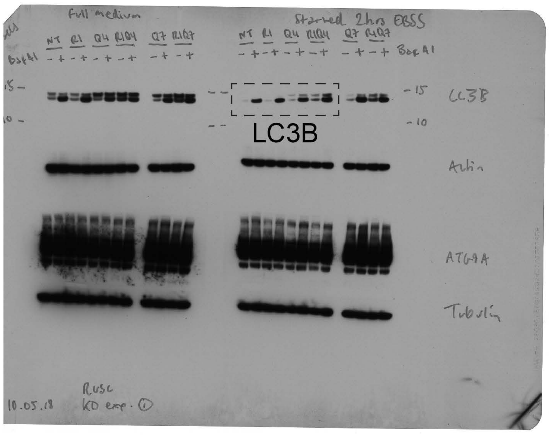

Fig. 8b: AP4B1

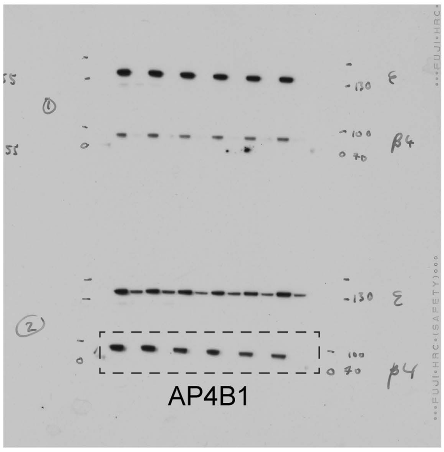

Fig. 8g: Actin

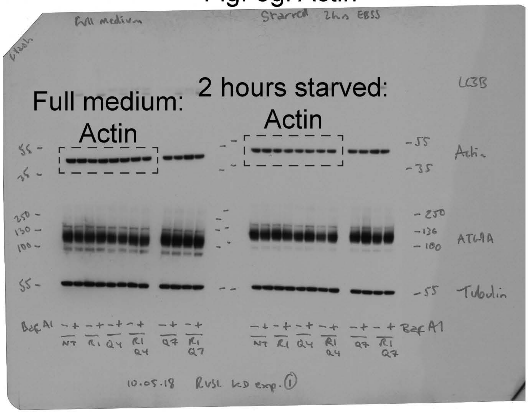

Supplementary Figure 11. Uncropped scans of Western blots shown in Fig. 8. The relevant lanes and the figures in which they appear are indicated on each blot.

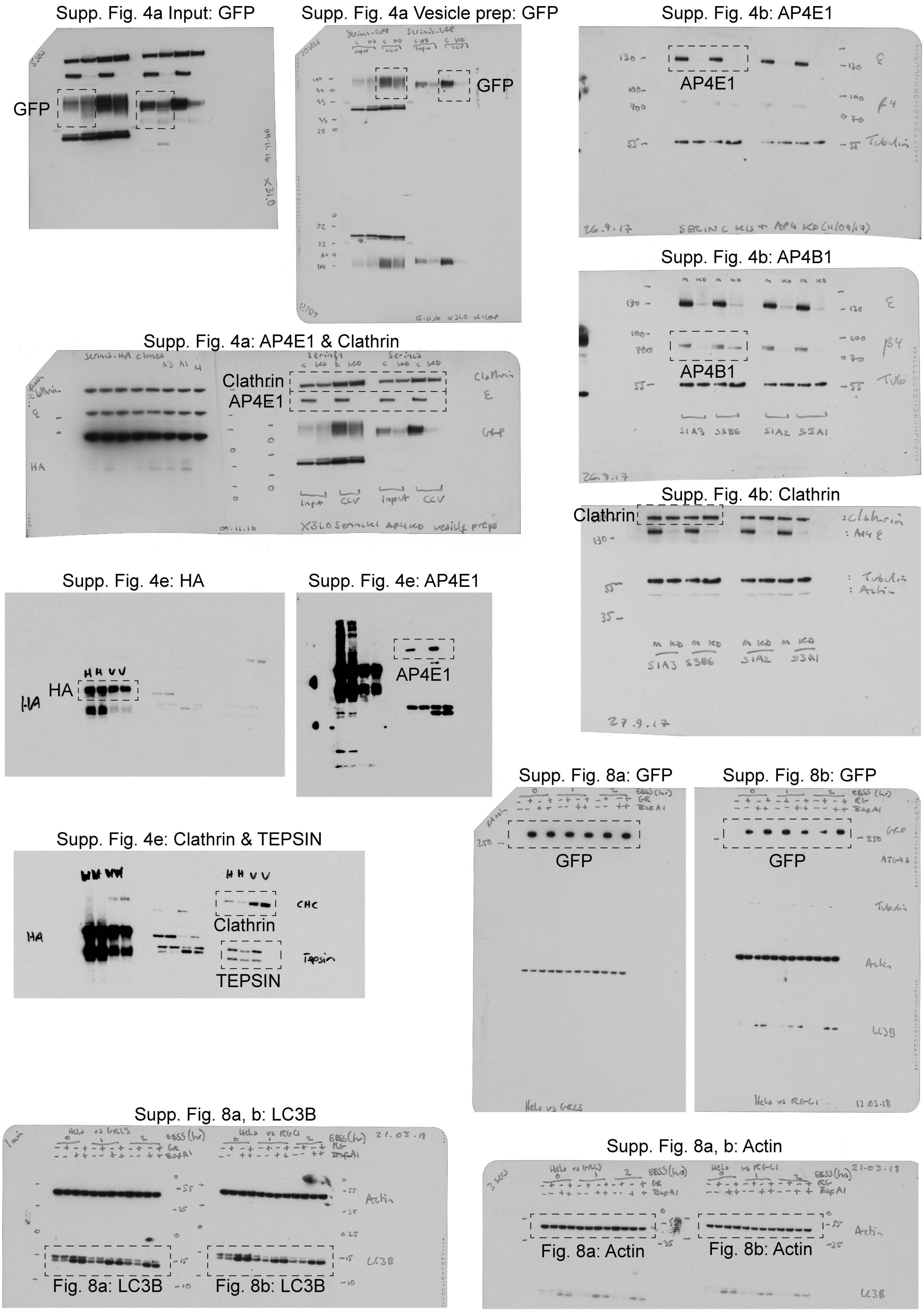

Supplementary Figure 12. Uncropped scans of Western blots shown in Supplementary Fig. 4 and 8. The relevant lanes and the figures in which they appear are indicated on each blot.

Supplementary Tables

Supplementary Table 1: Overview of mass spectrometric analyses

Sample	MS Quant Strategy	Number of replicates	Sample fractionation approach	MS instrument	Number of MS runs[1]
Dynamic organellar maps[2]	SILAC	2 x Wild-type	3 x SDB-RPS	Exactive HF	30
		2 x AP4E1 KO			30
		2 x AP4B1 KO			30
Whole cell lysates	SILAC	3 x Wild-type	6 x SCX	Exactive HF	18
		3 x AP4E1 KO			18
		3 x AP4B1 KO			18
Membrane fractions	LFQ	3 x Wild-type	3 x SDB-RPS	Exactive HF	9
		3 x AP4E1 KO			9
		3 x AP4B1 KO			9
Vesicle preparations	SILAC	3 x AP-4 KD	10 gel slices	Exactive	37[3]
		2 x AP-4 KS	10 gel slices	Exactive	20
		2 x AP4E1 KO	3 x SDB-RPS	Exactive HF	9[3]
		2 x APEB1 KO	3 x SDB-RPS	Exactive HF	6
Sensitive IPs	SILAC	3 x TEPSIN-GFP	1x SDB-RPS	Exactive HF	4[3]
Conventional IPs	SILAC	2 x TEPSIN-GFP	2 x 5 gel slices	Exactive	20
BioID	LFQ	3 x AP4B1-BirA*	1 x SDB-RPS	Exactive HF	3
		3 x AP4E1-BirA*			3
		3 x AP4M1-BirA*			3
		3 x AP4S1-BirA*			3
		3 x HeLa (control)			3
		3 x BirA* (control)			3
		3 x GFP-BirA* (control)			3
**Total number of MS samples**					**288**
**Total MS run time**					**30 days**

[1] Each run took approximately 150 minutes (HPLC gradient) + 25 minutes loading.

[2]Each map consisted of five samples (subfractions).

[3]Includes some technical replicates.

Supplementary Methods

Constructs

All primers used in the generation of the constructs used in this study are listed below. All constructs were generated using Gibson Assembly apart from pEGFP-myc-BirA* which was made using traditional restriction enzyme-based cloning.

pEGFP-myc-BirA* (cloning sites BsrGI and XbaI)
Amplification of myc-BirA* from pcDNA3.1_mycBioID:
F: CGGTAGCTGTACAAGATGGAACAAAAACTCATCT (contains BsrGI site)
R: GGTCGTTCTAGATCAGCGGTTTAAACTTAAG (contains XbaI site)

C-terminal myc-BirA* tagging construct (pLXINmod)
Amplification of myc-BirA* from pcDNA3.1_mycBioID:
F: TCTAGGCGCCGGAATTCGTTAGATCTGGCAGCGGCGGCAGCGGCAGCGGCATGGAACAAAAACTCATCTCAG (contains GS linker)
R: GATCCCTCGAGGTCGACGTTTTACTTCTCTGCGCTTCTCAG

AP4E1 BioID (pLXINmod_AP4E1_myc-BirA*)
Amplification of myc-BirA* from pcDNA3.1_mycBioID:
F: CCTGTACACCATGGAACAAAAACTCATCTC
R: GCAGAGCTCCGCGGTTTAAACTTAAGCTTG
Amplification of N-terminal part of AP4E1 (and linker) from pLXINmod_AP4E1_FKBP:
F: TCTAGGCGCCGGAATTCGTTGCGGCGATGAGCGACATA
R: TTTGTTCCATGGTGTACAGGTTCTTGGCGG
Amplification of C-terminal part of AP4E1 from pLXINmod_AP4E1_FKBP:
F: TTTAAACCGCGGAGCTCTGCCTGTTCCTC
R: GATCCCTCGAGGTCGACGTTCTAGGATCCCTCCATCACC

AP4B1 BioID (pLXINmod_AP4B1-myc-BirA*)
Amplification of *AP4B1* from Image clone 2906087:
F: CTAGGCGCCGGAATTCGTTAGCCACCATGCCGTACCTTGGCTCC
R: GCTGCCGCCGCTGCCAGATCCTGATTTTATTTCTTCAATTGTTCCAATC

AP4M1 BioID (pLXINmod_AP4M1-myc-BirA*)
Amplification of *AP4M1* from a sequence verified EST clone:
F: CTAGGCGCCGGAATTCGTTAGCCACCATGATTTCCCAATTCTTCATTCTG
R: GCTGCCGCCGCTGCCAGATCCGATCCGAATGACATAGGCG

AP4S1 BioID (pLXINmod_AP4S1-myc-BirA*)
Amplification of *AP4S1* from a sequence verified EST clone:
F: CTAGGCGCCGGAATTCGTTACATAACTTTTGAACTGTATTTGG
R: GCTGCCGCCGCTGCCAGATCCGCTTTCTGACATCTTATCAAG

pQCXIH_GFP-RUSC2

Amplification of *GFP* from pEGFP-N2:

F: GGAATTGATCCGCGGCCGCAGCCACCATGGTGAGCAAG

R: ACCGGTGCCAGATCCCTTGTACAGCTCGTCCATGC

Amplification of N-terminal part of *RUSC2* from pCMV-SPORT6_RUSC2:

F: GCTGTACAAGGGATCTGGCACCGGTGGCAGCGGCAGCGGCATGGATAGTCCCCCAAAGC

R: CCTGTTCCATGGAGCCGGTACTCAGTCAAG

Amplification of C-terminal part of *RUSC2* from pCMV-SPORT6_RUSC2:

F: TACCGGCTCCATGGAACAGGAAGCTTGC

R: AATTAAGCGTACGAGGCCTATCAGTTTTGGCTGCTTCC

pQCXIH_RUSC2-GFP

Amplification of *GFP* from pEGFP-N2:

F: CAGCCAAAACGGATCTGGCACCGGTGGCAGCGGCAGCGGCATGGTGAGCAAGGGCGAG

R: CGTACGAGGCCTACCGGTGCTTACTTGTACAGCTCGTCCATG

Amplification of N-terminal part of *RUSC2* from pCMV-SPORT6_RUSC2:

F: CGCTGCAGGAATTGATCCGCGGCCGCCACCATGGATAGTCCCCCAAAGC

R: CCTGTTCCATGGAGCCGGTACTCAGTCAAG

Amplification of C-terminal part of *RUSC2* from pCMV-SPORT6_RUSC2:

F: TACCGGCTCCATGGAACAGGAAGCTTGC

R: ACCGGTGCCAGATCCGTTTTGGCTGCTTCCAGG

pLXINmod_SERINC3_HA

Amplification of N-terminal part of *SERINC3* from pSERINC3-mCherry:

F: TCTAGGCGCCGGAATTCGTTACCGGTGCCACCATGGGGGCTGTG (contains AgeI site)

R: GGAACATCGTATGGGTACAGGGTTGGTGCAGTTATGCG

Amplification of C-terminal part of *SERINC3* from pSERINC3-mCherry:

F: ACCAACCCTGTACCCATACGATGTTCCAGATTACGCTGCTCCTGGAAATTCAACTGC (contains HA tag)

R: GATCCCTCGAGGTCGACGTTTTATTAGCTGAAGTCCCGACTGGTG

pDonor_myc-Clover_SERINC1 (SERINC1 endogenous tagging donor)

Amplification of 5' homology region of *SERINC1* from HeLa genomic DNA:

F: CTCCCCGGGCGCGACTAGTGAATTCGAGTGCAGTGGCTTGATC

R: GAGTTTTTGTTCAGAACCCGATCGGTCAAAATCACGATTTGTAAG

Amplification of 3' homology region of *SERINC1* from HeLa genomic DNA:

F: CATTATACGAAGTTATTTAATTAACAAGTGCATTGATATGTGAAGTAG

R: ATAATCAGCATCATGATGTGGTACCCTGCAACCTCTGCCTCCT

pDonor_myc-Clover_SERINC3 (SERINC3 endogenous tagging donor)

Amplification of 5' homology region of *SERINC3* from HeLa genomic DNA:

F: CTCCCCGGGCGCGACTAGTGAATTCTCCACCTCATGCTCTGCTT

R: GAGTTTTTGTTCAGAACCCGATCGGCTGAAGTCCCGACTGGTG

Amplification of 3' homology region of *SERINC3* from HeLa genomic DNA:

F: CATTATACGAAGTTATTTAATTAAGTGAATGCTTTGCAAGTTTG

R: ATAATCAGCATCATGATGTGGTACCGCATCCTGACTAGAAGCG

Supplementary References

1. Frazier, M. N. *et al.* Molecular Basis for the Interaction Between AP4 β4 and its Accessory Protein, Tepsin. *Traffic* **17,** 400–415 (2016).
2. Mattera, R., Guardia, C. M., Sidhu, S. S. & Bonifacino, J. S. Bivalent motif-ear interactions mediate the association of the accessory protein tepsin with the AP-4 adaptor complex. *J. Biol. Chem.* **290,** 30736–30749 (2015).
